# In Situ Fabrication of Cuprous Selenide Electrode via Selenization of Copper Current Collector for High‐Efficiency Potassium‐Ion and Sodium‐Ion Storage

**DOI:** 10.1002/advs.202104630

**Published:** 2021-12-23

**Authors:** Xi Chen, Malin Li, Shi‐Ping Wang, Chunzhong Wang, Zexiang Shen, Fu‐Quan Bai, Fei Du

**Affiliations:** ^1^ Key Laboratory of Physics and Technology for Advanced Batteries (Ministry of Education) State Key Laboratory of Superhard Materials College of Physics Jilin University Changchun 130012 P. R. China; ^2^ State Key Laboratory of Inorganic Synthesis and Preparative Chemistry College of Chemistry Jilin University Changchun 130012 P. R. China; ^3^ Laboratory of Theoretical and Computational Chemistry Institute of Theoretical Chemistry and College of Chemistry Jilin University Changchun 130012 P. R. China; ^4^ Division of Physics and Applied Physics School of Physical and Mathematical Sciences Nanyang Technological University Singapore 637616 Singapore

**Keywords:** cuprous selenides, electrode materials, potassium‐ion battery, sodium‐ion battery

## Abstract

Selenium‐based materials are considered as desirable candidates for potassium‐ion and sodium‐ion storage. Herein, an in situ fabrication method is developed to prepare an integrated cuprous selenide electrode by means of directly chemical selenization of the copper current collector with commercial selenium powder. Interestingly, only the electrolyte of 1 m potassium hexafluorophosphate dissolved in 1,2‐dimethoxyethane with higher highest occupied molecular orbital energy and lower desolvation energy facilitates the formation of polyselenide intermediates and the further selenization of the copper current collector. Benefiting from the unique thin‐film‐like nanosheet morphology and the robust structural stability of the integrated electrode, the volume change and the loss of selenide species could be effectively restrained. Therefore, high performance is achieved in both potassium‐ion batteries (462 mA h g^−1^ at 2 A g^−1^ for 300 cycles) and sodium‐ion batteries (775 mA h g^−1^ at 2 A g^−1^ for 4000 cycles). The facile fabrication strategy paves a new direction for the design and preparation of high‐performance electrodes.

## Graphical abstract

An in situ fabrication strategy is proposed to prepare the integrated Cu_2_Se electrode through selenization of Cu current collector by commercial selenium powder in a specific electrolyte (1 m KPF_6_ in 1,2‐dimethoxyethane). Benefiting from the thin‐film‐like nanosheet morphology and the robust structural stability, integrated Cu_2_Se electrode displays superior potassium‐ and sodium‐ion storage performance.

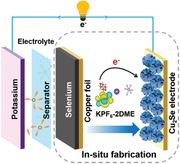

## 1 Introduction

Developing high‐performance energy storage devices is crucial for the effective utilization of renewable energy sources such as solar, wind, and tidal energy with the intermittent nature, securing the sufficient energy supply for sustainable development of modern society. Sodium‐ion batteries (SIBs) and potassium‐ion batteries (PIBs) are recognized as the competitive alternatives to lithium‐ion batteries (LIBs) for the next‐generation energy storage in consideration of sufficient reserves and low cost of the resources.^[^ ^1^, ^2^, ^3^ ^]^ However, the development of SIBs and PIBs has been hindered by the inherent sluggish reaction kinetics of the systems and the large volume variation of electrode materials during charge and discharge processes.^[^ ^4^, ^5^, ^6^ ^]^ Therefore, exploring electrode materials with high specific capacity, prominent rate capability, and superior cycling stability is of great significance to achieve high‐performance SIBs and PIBs.

Selenium‐based electrode materials, including selenium compounds and selenium‐containing composites, have attracted considerable attention owing to their high theoretical capacity and good electronic conductivity.^[^ ^7^, ^8^, ^9^, ^10^ ^]^ Nevertheless, the practical application of Se‐based electrode materials has still been hampered by two main issues. On the one hand, the huge volume fluctuation upon cycling gives rise to the agglomeration and the cracking of Se‐based electrodes, resulting in the severe degradation in performance.^[^ ^11^, ^12^, ^13^ ^]^ Extensive efforts have been made to buffer the volume change of Se‐based electrode via nanocrystallization,^[^ ^14^, ^15^, ^16^ ^]^ morphology regulation,^[^ ^17^, ^18^, ^19^, ^20^ ^]^ and hybrid composites construction,^[^ ^21^, ^22^, ^23^, ^24^ ^]^ which, unfortunately, might not be satisfactory for the practical production due to their complicated processes. On the other hand, the shuttle effect of the soluble intermediate polyselenides generated during the electrochemical processes of Se‐based electrodes would cause continuous loss of active materials, finally resulting in the irreversible capacity decay of batteries.^[^ ^25^, ^26^, ^27^ ^]^ To restrain the continuous loss of active selenium species, physical trapping and chemical bonding were proposed as the most effective approaches according to the present reports.^[^ ^11^, ^28^ ^]^ Specifically, the high order polyselenides could be physically confined in the host materials, typically porous carbon matrixes with various microstructure, such as fibers,^[^ ^29^ ^]^ tubes,^[^ ^30^ ^]^ spheres,^[^ ^31^ ^]^ nanosheets,^[^ ^32^ ^]^ etc. Besides, selenium species could also be chemically adsorbed by the matrix with manipulated surface properties. Zhang et al.^[^ ^33^ ^]^ developed a CoS_2_ decorated multi‐channeled carbon nanofibers host, in which the CoS_2_ particles served as polar sites to immobilize the intermediates and facilitate the redox kinetics. Ji's group^[^ ^34^ ^]^ fabricated a clew‐like Ni‐Pr/PPy spheres, in which the interface derived with Ni—O—C bonds led to the effective trapping of the polyselenides and highly reversible conversion reaction. Although the electrochemical performance was indeed improved via the above‐mentioned strategies, the host materials introduced to buffer the volume change or restrict the selenium loss might drastically decrease the gravimetric and volumetric capacities of Se‐based electrodes.

Herein, a chemically in situ fabrication method is proposed to prepare the integrated cuprous selenide (Cu_2_Se) electrode for SIBs and PIBs via the direct reaction between the commercial selenium powder and the copper current collector. Specifically, the selenium casting on the copper current collector tended to transform into soluble polyselenides spontaneously after being assembled in the battery with the ether‐based electrolyte at the early stage, followed by the further reaction with the copper foil to form Cu_2_Se after a relaxation period of 10 h. The comprehensive reaction mechanism was revealed via a series of external experiments combined with the theoretical calculation. The obtained Cu_2_Se electrode showed a self‐assembled nanosheet morphology, which is beneficial to the penetration of the electrolyte and the alleviation of the electrode volume expansion upon cycling. Moreover, the strong interaction between the selenium species and the electrode is favorable for the restriction of active materials loss. Benefiting from the improved reaction kinetics, the reduced volumetric expansion, as well as the mitigated side reactions, the in situ fabricated Cu_2_Se electrode demonstrated superior sodium and potassium storage performance (high reversible capacity of 775 and 462 mA h g^−1^ at 2 A g^−1^ for SIBs and PIBs, respectively). The facile and efficient fabrication strategy in the present work provides insights into the rational design and the practical production of the high‐performance selenium‐based electrodes for various energy storage devices.

## 2 Results and Discussion

### 2.1 Fabrication and Characterization of the Electrode

**Figure 1:**
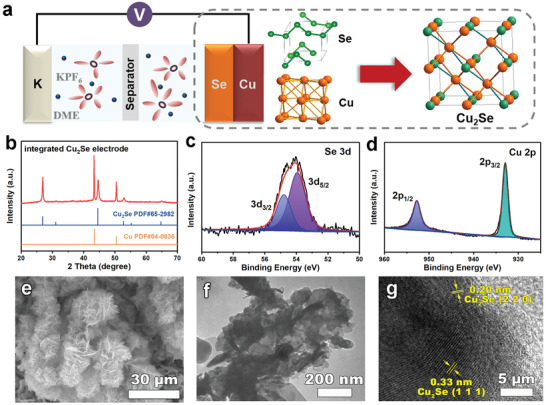
a) Schematic illustration of the battery structure and the fabrication process of the integrated Cu_2_Se electrode. b) XRD pattern of the integrated Cu_2_Se electrode with the standard XRD patterns of Cu_2_Se and Cu for identification. c) Se 3d and d) Cu 2p XPS spectra of the integrated Cu_2_Se electrode. e) SEM, f) TEM, and g) HRTEM images of the integrated Cu_2_Se electrode.

To prepare the precursory electrode, commercial selenium powder, Super P, and carboxyl methyl cellulose (CMC) binder were dispersed in deionized water with a weight ratio of 7:2:1, and then pasted on the rough side of copper foil to improve adhesion. After being dried at 80 °C for 12 h in vacuum, the precursory electrode with a mass loading of ≈1–1.5 mg cm^−2^ was obtained. X‐ray diffraction (XRD, Figure S1, Supporting Information) of the precursory electrode reveals mixed phases including Se and Cu, and the precursory electrode remained stable as confirmed by the X‐ray photoelectron spectroscopy (XPS, Figure S2, Supporting Information). Then, the integrated cuprous selenide electrode was prepared by assembling the cell using the precursory electrode and the potassium foil with the electrolyte of 1 m potassium hexafluorophosphate (KPF_6_) dissolved in 1,2‐dimethoxyethane (DME), as illustrated in **Figure** **1**a. After relaxing for 10 h, Cu_2_Se was dramatically formed via the gradually chemical reactions between Se and Cu foil with the impact of specific electrolyte accompanied by the complete vanish of selenium. As shown in Figure 1b, all the XRD reflections of the as‐obtained electrode can be indexed on the basis of the cubic Cu_2_Se (PDF#65‐2982) with a space group of Fm−3m. Furthermore, the Se 3d high‐resolution XPS spectrum of the electrode (Figure 1c) exhibits two peaks centered at 53.9 and 54.8 eV, corresponding to Se^2−^ 3d_5/2_ and Se^2−^ 3d_3/2_ for Cu_2_Se. As for the Cu 2p spectrum (Figure 1d), two peaks centered at around 932.9 and 952.7 eV are consistent with 2p_3/2_ and 2p_1/2_ of Cu^+^, respectively

**Figure 2:**
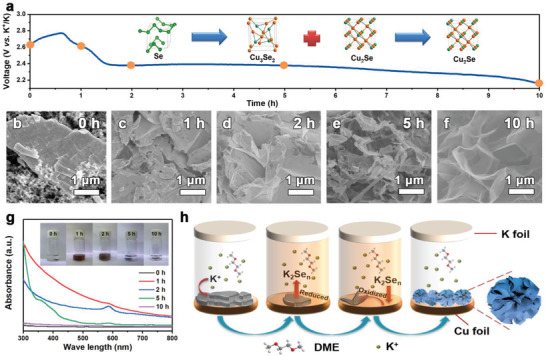
a) The voltage variation of the as‐assembled battery using precursory electrode during the relaxation period. b–f) SEM images of the electrodes relaxed for 0, 1, 2, 5, and 10 h. g) UV–vis absorption spectra of the DME solution used to immerse the electrodes relaxed for 0, 1, 2, 5, and 10 h. The inset of (g) presents the digital pictures of the solution used to immerse the electrodes relaxed for 0, 1, 2, 5, and 10 h. h) Schematic illustration of reaction procedure on the electrode upon relaxation period.

The low magnification scanning electron microscopy (SEM) image shows that the electrode presented a cluster‐like morphology assembled by the thin‐film‐like nanosheets (Figure 1e). Particularly, the nanosheets interweaved to form a nanoporous network (**Figure** **2**f), which can be further revealed by the transmission electron microscopy (TEM) image (Figure 1f). Such unique structure could not only effectively facile the electrolyte infiltration and the charge transfer, but also significantly relieve the volume variation upon cycling, ensuring the superior electrochemical performance of batteries. Energy‐dispersive X‐ray spectroscopy mapping (Figure S3e, Supporting Information) confirms a uniform distribution of Cu and Se elements in the electrode. Furthermore, the high‐resolution TEM (HRTEM) image (Figure 1g) displays a series of lattice fringes with the spacing distance of 0.20 and 0.33 nm, corresponding to the (220) and (111) planes of Cu_2_Se. This result is in accordance with the XRD pattern (Figure 1b) and the XPS spectra (Figure 1c,d), demonstrating the successful chemically in situ fabrication of the integrated Cu_2_Se electrode.

### 2.2 Evolution Processes of the Integrated Electrode

The chemically in situ fabrication progress of the integrated Cu_2_Se electrode was preliminarily embodied in the voltage variation during the relaxation period. As shown in Figure 2a, the voltage of the assembled cell with precursory electrode was measured to be 2.65 V at the initial stage, which experienced a small rise and then dropped below 2.20 V during the 10‐h period of relaxation. Such a voltage variation implies the evolution of phases, morphology, and interfacial properties for the electrode during the relaxation period. To gain further understanding on the formation processes of the integrated Cu_2_Se electrode, the morphology variation and the phase evolution of the electrode at different relaxation stages have been comprehensively investigated. To begin with, SEM was employed to observe the morphology variation of the electrode. The bulk Se with a smooth surface in the precursory electrode (Figure 2b) tended to split into small pieces after the cell was relaxed for an hour (Figure 2c). With the prolongation of relaxation period, the pieces gradually converted into nanoplates with a decreasing thickness, and finally formed the interconnected ultrathin nanosheets (Figure 2d–f). Furthermore, the content of Cu in the electrode material gradually increased with the relaxation period (Figure S3, Supporting Information), while Cu and Se elements uniformly distrib uted in the final product when the integrated Cu_2_Se electrode was obtained after 10 h.

Subsequently, the phase evolution was monitored via XRD and UV–vis absorption spectroscopy combined with the external experiment analysis (see Experimental Section for details). As shown in Figure S4, Supporting Information, the electrode was composed of the trigonal Se at the beginning of the reaction process. After being relaxed for an hour, several new diffraction peaks corresponding to the tetragonal Cu_3_Se_2_ and cubic Cu_2_Se emerged in the XRD pattern of the electrode except for the peaks from the original Se (Figure S4, Supporting Information). Aiming to give an insight into the reaction processes, the electrode that relaxed for 1 h was fetched out from the cell and then immersed in DME. The color of the solution turned dark brown after adding the electrode (inset of Figure 2g), probably due to the formation of soluble potassium polyselenide intermediates during the first hour relaxation. Then, the solution was detected via UV–vis absorption spectroscopy measurement to identify the specific ingredients. A broad peak emerged at around 587 nm indeed demonstrated the formation of soluble polyselenides (Figure 2g), which could easily react with Cu to form copper selenides.^[^ ^35^ ^]^ The reduction of selenium and the selenization of Cu foil continuously proceeded after the cell being relaxed for 2 h, maintaining the coexistence of Se, Cu_2_Se, and Cu_3_Se_2_ (Figure 2g and Figure S4, Supporting Information). After a 5‐h relaxation, it seems that most of the polyselenides reacted with the Cu foil as the DME solution soaking the electrode is almost colorless (inset of Figure 2g). However, the UV–vis spectrum and the XRD pattern are suggestive of an incomplete reaction on the electrode (Figure 2g and Figure S4, Supporting Information), though the cuprous selenide was the major ingredient of the electrode. Eventually, the reaction went to completion after the cell was relaxed for 10 h, all the Se reacted with the Cu foil to form cuprous selenide (Figure S4, Supporting Information).

Additionally, the electrodes with different relaxation periods exhibited diverse electrochemical properties according to their galvanostatic charge/discharge (GCD) profiles and the corresponding differential capacity versus voltage (d*Q*/d*V*) curves, which provided further evidence for the phase evolution from another perspective (Figure S5, Supporting Information). As shown in Figure S5a–c, Supporting Information, the fresh battery with no relaxation period exhibited a redox pair at around 2.2 V in the first cycle, which could be assigned to the potassiation and depotassiation processes of selenium.^[^ ^36^ ^]^ The intensity of these redox peaks significantly reduced accompanied by the appearance of new redox pairs for the battery with a relaxation period of 1 h (Figure S5d–f, Supporting Information), indicating the phase transition of the precursory electrode so as to change the charge and discharge behavior of the battery. With the extension of relaxation period, the new redox pairs were intensified, and the capacity of the battery was increased simultaneously. In contrast, the redox for the (de)potassiation of selenium dwindled, corresponding to the continuous consumption of the pristine Se. Finally, the redox potential for the electrode with a relaxation period of 10 h accorded exactly with that of the (de)potassiation for cuprous selenide (Figures S5m–o and S6, Supporting Information), illustrating the successful fabrication of the integrated cuprous selenide electrode. Notably, both the capacity and the redox intensity increased with the prolonging relaxation period of the batteries in the second cycle, demonstrating the better reversibility of the integrated cuprous selenide electrode. Based on the above analysis, the evolution process of the electrode can be summarized as shown in Figure 2h. After being assembled in the battery, the bulk selenium on the electrode gradually transforms into soluble polyselenides. Then, the polyselenides infiltrate from the crack of selenide, and react with the copper current collector to form a series of selenides such as Cu_3_Se_2_ and Cu_2_Se. As the reaction progress, the integrated Cu_2_Se electrode with nanosheet‐like morphology can be obtained on account of the reducibility of selenium species.

### 2.3 Key Factors for the Selenization of the Cu Current Collector

**Figure 3:**
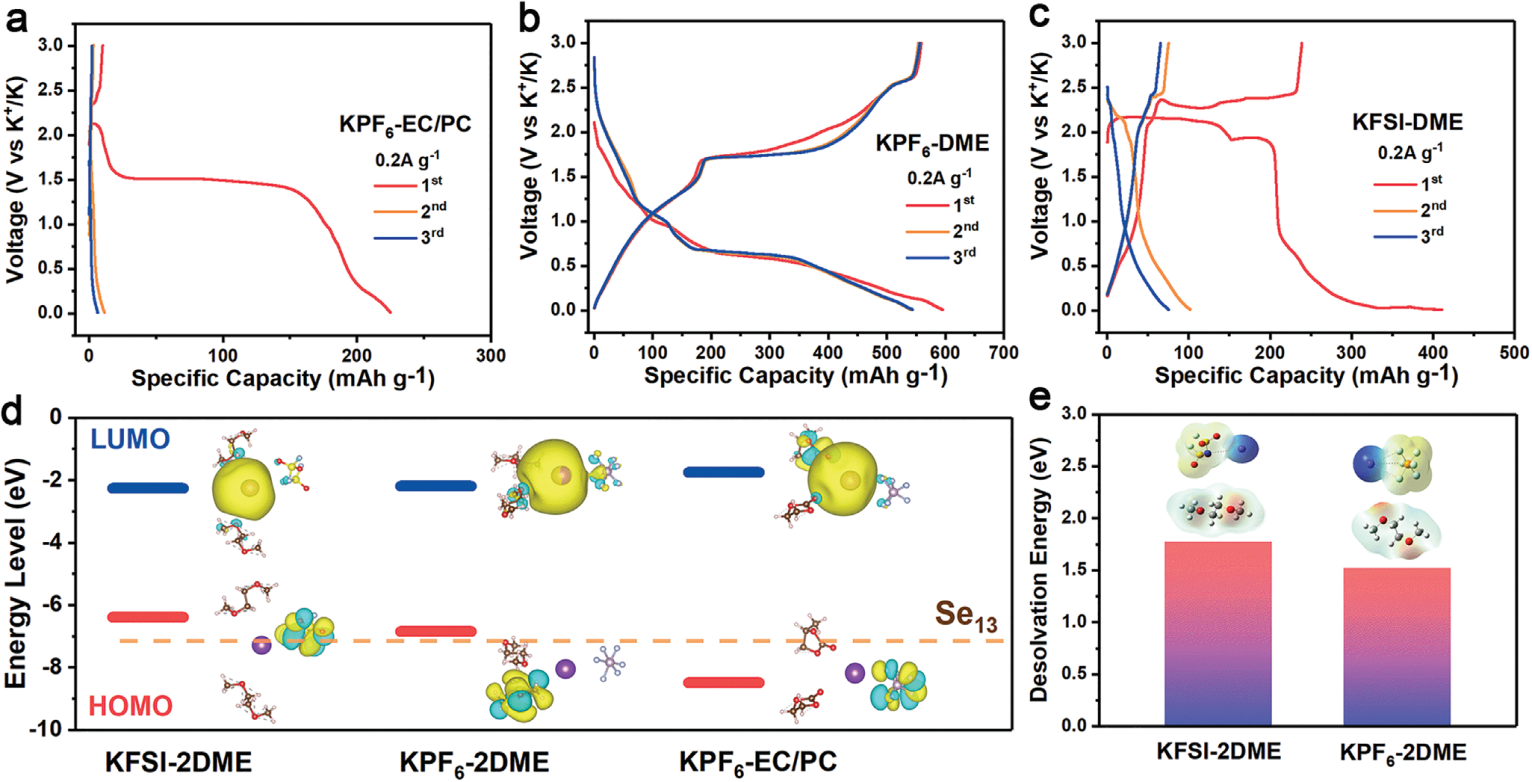
The GCD profiles of the batteries using different electrolytes: a) 1 m KPF_6_ in EC/PC, b) 1 m KPF_6_ in DME, and c) 1 m KFSI in DME. d) The highest occupied molecular orbital (HOMO) and the lowest unoccupied molecular orbital (LUMO) energy levels of salt‐solvent complexes for the three different electrolytes (inset: the corresponding molecular orbital profiles). e) Desolvation energies of KFSI‐2DME and KPF_6_‐2DME, as well as the characteristics of eletrostatic potential.

Notably, since the assembled battery was at an open circuit state during the relaxation period, the generation of intermediate polyselenides and the selenization of the copper current collector originated from the chemical reactions on the surface of the electrode rather than the electrochemical reactions, and related to the specific electrolyte (1 m KPF_6_ dissolved in DME). When the electrolyte solvent was replaced with ethylene carbonate (EC)/propylene carbonate (PC) with a volume ratio of 1:1, the GCD profiles of the battery (**Figure** **3**a) were totally different from those of the battery using 1 m KPF_6_ dissolved in DME after a 10‐h relaxation (Figure 3b). The only plateau presented during the discharge process derives from the single phase‐change reaction of Se in the carbonate‐based electrolytes.^[^ ^37^ ^]^ While the lower capacity and the charging irreversibility originate from the large particle size of the commercial Se chain, the high reaction barrier of the one‐step solid reaction, as well as the side reaction between the discharge product K*_x_* Se and the carbonate‐based electrolyte. The XRD pattern also corroborates that the selenium in the precursory electrode remained unchanged after being relaxed in 1 m KPF_6_ in EC/PC for 10 h (Figure S7, Supporting Information). Therefore, the employment of carbonate‐based electrolyte could not induce the in situ fabrication of the integrated Cu_2_Se electrode in spite of using KPF_6_ as the salt. Besides, the salt of the electrolyte also makes a considerable impact on the entire system. For comparison, the electrolyte was prepared by substituting potassium bis(fluorosulfonyl)imide (KFSI) for KPF_6_ in DME solvent. As shown in Figure S7, Supporting Information, the electrode maintained the original selenium phase after being relaxed in the electrolyte of 1 m KFSI in DME for 10 h. In addition, the initial charge and discharge behavior of the battery with 1 m KFSI in DME as the electrolyte was in agreement with the two‐phase transition process of Se electrode in ether‐based electrolyte (Figure 3c),^[^ ^36^, ^38^ ^]^ and significantly deteriorated upon cycling due to the sluggish kinetics of Se electrode and the continuous loss of the Se species in ether‐based electrolyte. In consequence, the integrated Cu_2_Se electrode could only be in situ fabricated using specific electrolyte (1 m KPF_6_ in DME) so as to achieve superior electrochemical performance.

In order to gain a better understanding of the electrolyte selectivity to fabricate the integrated Cu_2_Se electrode, an interfacial model based on one potassium ion coordination structure was introduced to illustrate the in situ selenization process of the current collector via density functional theory (DFT) calculations. Generally, the solvated structure with a lowest energy is the main species in the electrolyte. Specifically, the cations coordinate with the solvent molecules in the first solvation shell, and the anions stay around the solvation shell with a certain distance from the cations in the diluted electrolyte (e.g., a concentration of around 1 m).^[^ ^39^, ^40^, ^41^ ^]^ The optimized geometrical structures of salt–solvent complexes for the above‐mentioned three different electrolytes are presented in Figure 3d. The highest occupied molecular orbital (HOMO) serves as the critical orbitals of the K^+^−solvent−anion complex, where the higher the energy level, the more conducive to losing electrons. Additionally, Se_13_ cluster was extracted from the trigonal selenium structure as the most stable unit (Figure S8, Supporting Information) to evaluate the interaction between the precursory selenium electrode and the electrolyte. According to the energy levels of the salt‐solvent complexes and the Se_13_ cluster, it is obvious that the HOMO energy of KPF_6_‐EC/PC is much lower than those of KFSI‐2DME, KPF_6_‐2DME and even Se_13_ cluster, so the reduction of bulk selenium to polyselenide would hardly occur in the carbonate‐based electrolyte. As for the ether‐based electrolyte systems, although the KFSI‐2DME system possesses a higher HOMO energy, which is preferable for the generation of polyselenide (Figure S9, Supporting Information), its higher desolvation energy (Figure 3e) probably renders a robust interaction between the polyselenide species and the solvated structure, which prevents the further selenization of Cu foil. These results interpret why the in situ fabrication of the integrated Cu_2_Se electrode could only take place in the specific electrolyte system (1 m KPF_6_ in DME). Besides, it is worth noting that, owing to such an anion‐induce effect, the soluble selenium species generated during the electrochemical process in KFSI/DME electrolyte would not react with Cu current collector, causing the fast capacity degradation as shown in Figure 3c. In addition, the cation of the electrolyte salt could impact the selenization process. When assembled the precursory electrode in a SIB with an electrolyte of 1 m sodium hexafluorophosphate (NaPF_6_) in DME, the coexistence of Se, intermediate Cu_3_Se_2,_ and Cu_2_Se was observed even after a relaxation time of 10 h (Figure S10, Supporting Information). It demonstrates a slower evolution kinetics of the electrode in SIB compared with that in PIB, probably due to the stronger interaction between the solvent molecular and sodium ions, which possess higher Lewis acidity than potassium ions.^[^ ^42^ ^]^ Accordingly, the in situ fabrication of the integrated Cu_2_Se electrode might be achieved in other battery systems via electrolyte regulation, paving a new way for the design and the preparation of the electrode.

Except for the electrolyte, the mass loading would also affect the fabrication process of the integrated Cu_2_Se electrode. The precursory electrodes with different mass loading (≈0.5–0.75 and ≈2.0–3.0 mg cm^−2^) were produced for comparison. In the case of electrode with lower mass loading, the selenization of Cu current collector was faster with a shorter existing time of the intermediate Cu_3_Se_2_ (Figure S11, Supporting Information). It is ascribed with the shorter diffusion path of the polyselenides on the precursory electrode with lower mass loading, corresponding to an easy access of copper. By contrast, the precursory electrode with higher mass loading exhibited an incomplete conversion even after a 10‐h relaxation. Particularly, the diffraction peaks of Cu_3_Se_2_ remained after 10 h as shown in Figure S12, Supporting Information, indicating the slower diffusion kinetics of the polyselenides toward copper current collector due to the higher mass loading. Therefore, a proper mass loading of the precursory electrode is of significance for the complete selenization of the copper current collector.

Furthermore, the copper current collector with high affinity toward Se is another key factor to fabricate the integrated Se‐based electrode. For comparison, aluminum (Al), nickel (Ni), and titanium (Ti) foils were also employed as the current collector, and the corresponding precursory electrodes were prepared in the same way (seen details in Experimental Section). After a 10‐h relaxation, the electrodes with different current collectors were also fetched out from the batteries using 1 m KPF_6_ in DME as the electrolyte, and then immersed in DME solvent. The DME solvent displayed a color of brown for all three control groups (Figure S13, Supporting Information), suggesting the existence of unreacted polyselenides. The XRD patterns also demonstrate that Se completely maintains its original phase without any changes for the electrodes with Al, Ni, and Ti as the current collector (Figure S14a–c, Supporting Information). The GCD profiles (Figure S14d–f, Supporting Information) of the batteries using these three current collectors are consistent with the intrinsic galvanostatic charge and discharge curves of Se, which has been illustrated in Figure S5a, Supporting Information. Thus, the copper current collector with unique selenophilic nature is indispensable for the in situ fabrication of integrated electrode.

### 2.4 Potassium Ions Storage Behavior

**Figure 4:**
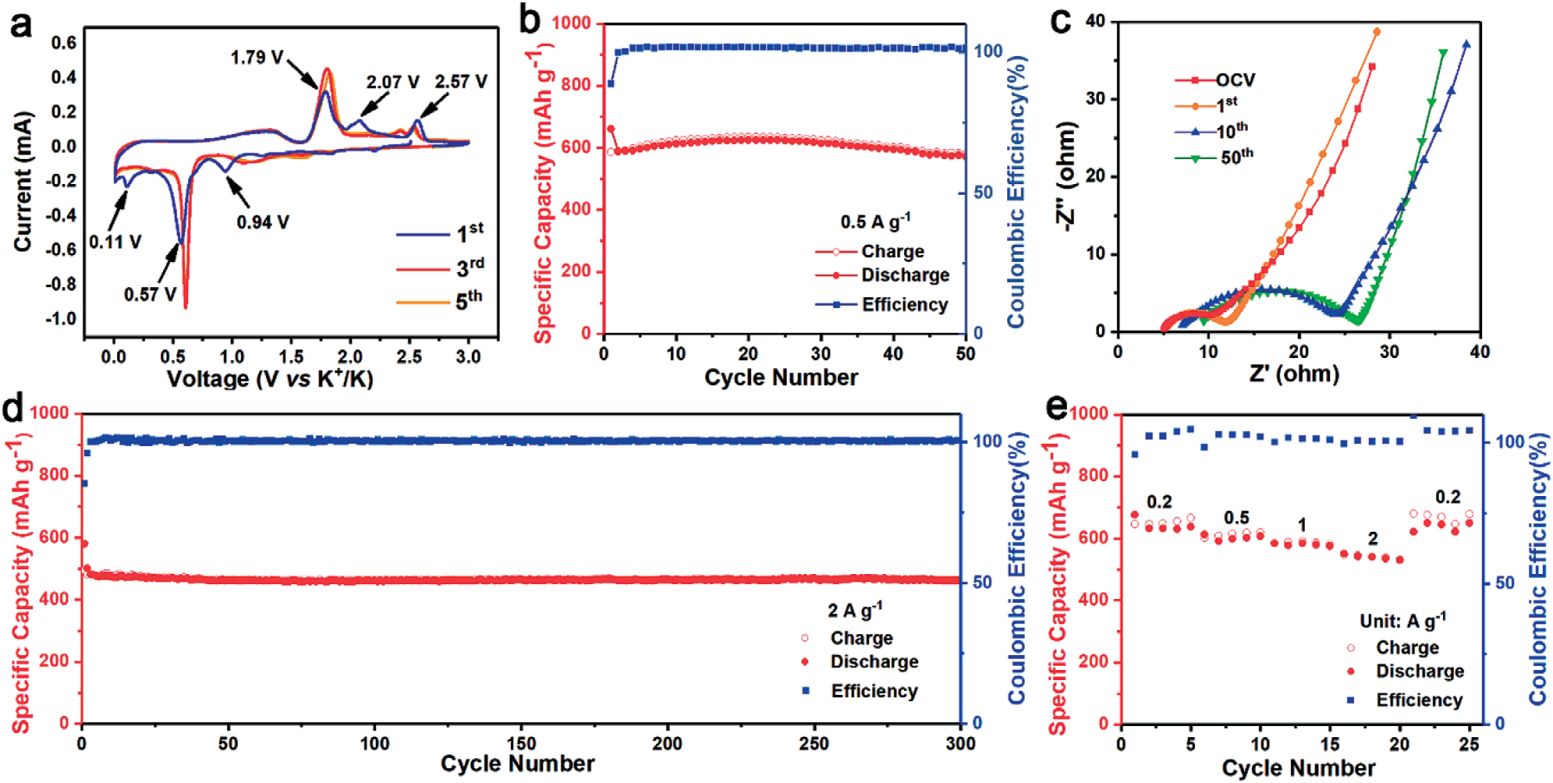
The electrochemical properties of the integrated Cu_2_Se electrode with the electrolyte of 1 m KPF_6_ in DME: a) CV curves for the first, third, and fifth cycle at a scan rate of 0.1 mV s^−1^ in a voltage range from 0.01 to 3.0 V (versus K/K^+^); b) the cycling performance at 0.5 A g^−1^; c) Nyquist plots of the battery after various cycles in a frequency range from 1 MHz to 1 mHz; d) the long‐term cycling performance at 2 A g^−1^; and e) the rate performance at various current densities.

According to Figure 3b, the integrated Cu_2_Se electrode delivered a high specific capacity of 548 mA h g^−1^ with superior cyclic reversibility. The GCD profiles with several plateaus and inflections demonstrate a multi‐step potassiation/de‐potassiation process, which can be observed more clearly through the cyclic voltammetry (CV) test (**Figure** **4**a). In the first cycle, two obvious peaks appearing at 0.94 and 0.57 V in the cathodic scan can be assigned to the insertion of potassium ions into the lattice of Cu_2_Se followed by the conversion reaction to form copper and potassium selenides. In addition, a small cathodic peak at 0.11 V, which disappeared after the first scan, can be attributed to the formation of solid electrolyte interface (SEI) film. Upon the initial anodic process, a series of redox peaks emerged in the voltage range from 1.3 to 2.6 V, representing the multiple depotassiation stages including conversion reaction (1.79 V) and extration reaction (2.07 and 2.57 V). Notably, the cathodic/anodic curves for the subsequent scans are almost coincident, displaying a good reversibility and stability during repeatable potassiation and depotassiation. The slight shift of the redox peaks in comparison with the first scan is attributed to some irreversible structure changes, such as the microstructure and the surface/interface properties of the materials, which may facilitate the reversible electrochemical reaction and ensure the fast redox kinetics in subsequent cycles.^[^ ^43^, ^44^ ^]^

Figure 4b shows the cyclic performance of the integrated Cu_2_Se electrode at a current density of 0.5 A g^−1^. The discharge capacity of the battery was 661 mA h g^−1^ (based on the active material of Se) for the first cycle, and remained almost unchanged after 50 cycles with a highly capacity retention of 98.8%. The Coulombic efficiency for the initial cycle was ≈88.7%, which is slightly lower due to the irreversible reactions, and increased above 99.8% after the second cycle. The XRD pattern of the integrated Cu_2_Se electrode after being cycled demonstrates the superior reversibility of the Cu_2_Se electrode (Figure S15, Supporting Information), corresponding to the high Coulombic efficiency upon cycling. Subsequently, the electrochemical impedance spectroscopy spectra (EIS) of the integrated Cu_2_Se electrode were collected after various cycles to estimate the electrode evolution upon cycling. As shown in Figure 4c, all the Nyquist plots consist of a single semicircle with an oblique line. The depressed semicircle is related to the charge‐transfer resistance (*R*_ct_) at the interface between the electrolyte and the electrode, while the oblique line is interpreted as the Warburg resistance reflecting the potassium‐ion diffusion in the bulk electrode.^[^ ^45^ ^]^ As shown in Table S1, Supporting Information, the *R*_ct_ value for the first cycle is slightly higher than that at the open‐circuit voltage (OCV) state, which correlates to the formation of SEI film. After 10 cycles, the *R*_ct_ increased due to the activation process of the electrode materials, and basically remained on the same order of magnitude after 50 cycles, ensuring the fast kinetic of the battery to achieve stable cycling performance. Furthermore, the long‐term cycling stability of the integrated Cu_2_Se electrode was estimated at a current density of 2 A g^−1^ (Figure 4d). A reversible capacity of 462 mA h g^−1^ was achieved over 300 cycles with a stable Coulombic efficiency of nearly 100%. The impressive cyclic performance might arise from the unique interconnected ultrathin nanosheet morphology of the integrated Cu_2_Se electrode, which could not only effectively facile reaction kinetics, but also relieve the volume variation of the conversion‐type materials. While the ex situ SEM images confirm the morphology integrity of electrode with a nanoporous network after cycling (Figure S16, Supporting Information). Except for the interconnected ultrathin nanosheet morphology, the robust adhesive force of the active materials on the current collector, which is attributed to the in situ chemical fabrication process rather than the binder adhesion, is also beneficial for the prolonged cycle life of the battery. Benefiting from the rational design of the integrated electrode structure, the integrated Cu_2_Se electrode also exhibited superior rate capability. As shown in Figure 4e, the integrated Cu_2_Se electrode displayed high reversible capacities of 647, 598, 588, and 541 mA h g^−1^ at 0.2, 0.5, 1, and 2 A g^−1^, respectively. Even at a high rate of 2 A g^−1^, it still delivered an 84% capacity retention with respect to 0.2 A g^−1^. Besides, the reversible capacity recovered to 644 mA h g^−1^ as the current density returned to 0.2 A g^−1^, indicating excellent structure stability of the integrated Cu_2_Se electrode.

### 2.5 Sodium Ions Storage Behaviors

**Figure 5:**
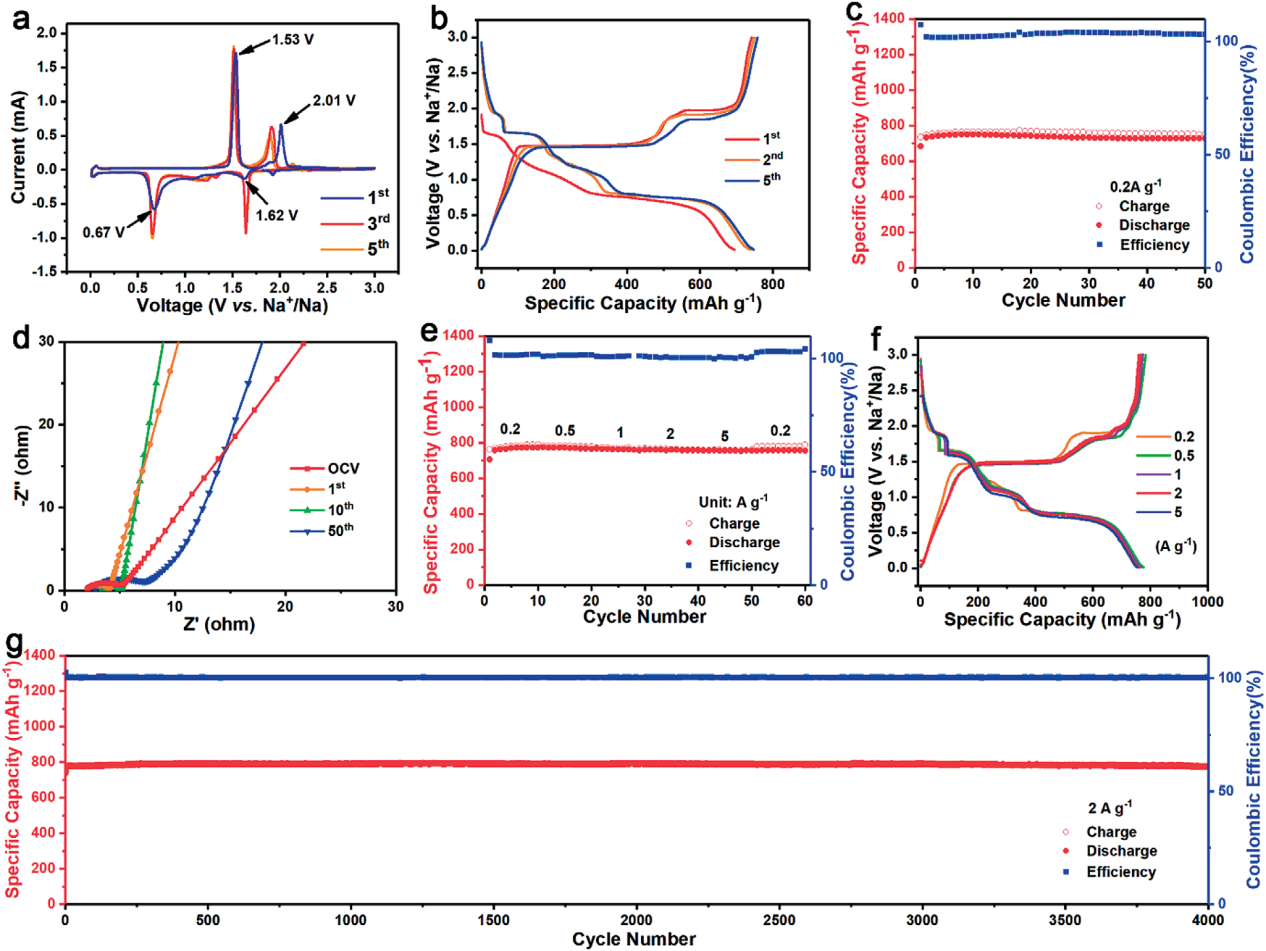
The electrochemical properties of the integrated Cu_2_Se electrode with the electrolyte of 1 m NaPF_6_ in DME: a) CV curves for the first, third, and fifth cycle at a scan rate of 0.1 mV s^−1^ in a voltage range from 0.01 to 3.0 V (versus Na/Na^+^); b) the GCD profiles at a current density of 0.2 A g^−1^; c) the cycling performance at 0.2 A g^−1^; d) Nyquist plots of the battery after various cycles; e) the rate performance and f) the corresponding GCD curves at various current densities; g) the long‐term cycling performance at 2 A g^−1^.

To extend the utilization of the integrated Cu_2_Se electrode, the sodium‐ion storage performance of the in situ fabricated electrode was comprehensively investigated using a corresponding ether‐based electrolyte of 1 m NaPF_6_ in DME. The detailed assembling process of the SIB is provided in the Experimental Section. The electrochemical process of the integrated Cu_2_Se electrode in SIBs is analogous with that in PIBs, including the insertion/extraction and conversion reactions as shown in CV curves (**Figure** **5**a). In the initial cathodic scan, the peak emerged at 1.62 V, which intensified in the subsequent scans, with some small humps between 1.7 and 1.0 V represents the insertion of sodium ions in the Cu_2_Se. The obvious peak at 0.67 V belongs to the conversion reaction to form metal Cu and Na_2_Se. The increasing intensity of cathodic peaks in the subsequent scans originated from the activation of the electrode after a reversible Na^+^ intercalation‐deintercalation process during the initial scan. In the initial anodic scan, two oxidation peaks around 1.53 and 2.01 V are attributed to the desodiation process generating the Cu_2−_ *_x_* Se structure with lattice vacancy. The structure evolution with the formation of lattice vacancy might be responsible for the peak shifting in the subsequent scans. Figure 5b presents the GCD profiles of the integrated electrode, which is in accordance with the CV curves. Benefiting from the unique morphology and the microstructure, the Cu_2_Se electrode delivered a highly reversible specific capacity of 743 mA h g^−1^ (Figure 5c), which retained to be 727 mA h g^−1^ after 50 cycles with a corresponding retention of 98%. The excellent cycling stability is due to the high reversibility of the integrated Cu_2_Se electrode (Figure S17, Supporting Information). To gain insight into the origin of the fascinating electrochemical performance, EIS was conducted after various cycles as shown in Figure 5d. The impedance of the battery did not increase significantly after 50 cycles (Table S2, Supporting Information), revealing the great stability of the electrode structure so as to achieve superior cycling performance. Additionally, the fast kinetics is favorable for the rate performance of the integrated Cu_2_Se electrode (Figure 5e). As the current density increased from 0.2 to 5 A g^−1^, the specific capacity of the battery was preserved to be 755 mA h g^−1^, only 2.1% decrease compared with the capacity at 0.2 A g^−1^. Moreover, the GCD profiles of the integrated Cu_2_Se electrode at various current densities overlap well without significant increase of overpotential (Figure 5f). Such an unprecedented rate performance is not only because of the unique structure of the integrated electrode which could facilitate the fast charge transfer during electrochemical process, but also due to the enhancement of conductivity derived from the structure vacancy and sodium‐ion insertion.^[^ ^46^, ^47^ ^]^ Encouraged by this fast sodium ion storage performance, the long‐term cycling capability of the integrated Cu_2_Se electrode was estimated at a high rate of 2 A g^−1^ as shown in Figure 5g. During the cycling process, the specific capacity of the integrated Cu_2_Se electrode remained stable even with no significant fluctuation, demonstrating a ≈100% capacity retention after 4000 cycles. The prominent electrochemical performance is ascribed to the rational design of the integrated Cu_2_Se electrode with the interconnected ultrathin nanosheets morphology and the robots interaction between the active materials and the current collector. Different from the negative effect of the reaction between the current collector and the active materials in the conventional battery systems, the in situ selenization of the copper current collector in this work emerges as a facile and effective approach to fabricate an integrated Cu_2_Se electrode with enhanced electrochemical performance. On one hand, the in situ chemical fabrication strategy partially converts the copper current collector, that is inert in other battery systems, into a component of active materials. It is equivalent to increasing the ratio of active material in the electrode while keeping the total mass of the electrode constant, which is of considerable importance to achieve a high energy density of the battery. On the other hand, the unique structure endows the electrode with the capabilities to facilitate the mass transition, buffer the volume change, and prevent the loss of active materials. Thus, the in situ fabricated Cu_2_Se electrode displays exceptional performance for potassium‐ and sodium‐ion storage, and possesses the potential for practical application.

## 3 Conclusion

In summary, a facile in situ fabrication method is proposed to prepare an integrated Cu_2_Se electrode through the directly chemical selenization of the copper current collector with commercial selenium powder. During the in situ selenization process, the composition of electrolyte plays a key role in the formation of polyselenide intermediates and the further selenization of the copper current collector. The as‐obtained integrated Cu_2_Se electrode with interconnected nanosheet morphology is not only favorable to facilitate the transmission of electrolyte and charge carriers, but also beneficial to alleviate the volume expansion during the conversion reaction. As a result, the integrated Cu_2_Se electrode exhibits superior sodium‐ion (775 mA h g^−1^ at 2 A g^−1^ for 4000 cycles) and potassium‐ion storage performance (462 mA h g^−1^ at 2 A g^−1^ for 300 cycles). The facile in situ fabrication strategy provides guidance for the design and preparation of high‐performance electrodes for practical application.

## 4 Experimental Section

### Chemicals and Materials

All the chemicals were of analytical grade and directly used. Selenium powder, CMC, DME were all purchased from Sigma Aldrich. Cuprous selenide was purchased from Aladdin Reagent.

### Fabrication of Electrodes

Commercial Se powder (or cuprous selenide), Super P conductive additive, and CMC binder with a weight ratio of 7:2:1 were dissolved in deionized water to prepare the slurry casting on the current collectors (Cu, Ni, Ti, and Al foil). After being dried in a vacuum oven at 80 °C for 12 h, the as‐prepared electrodes were punched into discs with the diameter of 10 mm. The mass loading of the active material was measured to be ≈1–1.5 mg cm^−2^.

### External Experiments

The electrodes that relaxed for 0, 1, 2, 5, 10 h were fetched out from the batteries and then immersed in DME solvent, which has usually been employed as the solvent to dissolve polyselenides.

### Materials Characterization

The crystal structure of the sample was studied by XRD measurement on a Bruker D8 Advance Diffractometer with Cu K*_*α*_* radiation. The morphologies and the microstructures of the samples were investigated using a NOVA 230 field‐emission scanning electron microscopy and a FEI Titan 80–300 HRTEM. X‐ray photoelectron spectra were tested by using a VG scientific ESCALAB‐250 spectrometer. UV–vis absorption spectra were conducted on a Shimadzu UV‐1900 spectrophotometer.

### Electrochemical Measurement

The electrochemical properties of the electrodes were evaluated by assembling 2032‐type coin cells in the argon filled glovebox. Potassium and sodium foils served as the counter electrodes for PIBs and SIBs, respectively. Glass fiber filter (Whatman GF/F for PIBs and GF/C for SIBs) was employed as the separator. For PIBs, 1 m KPF_6_ dissolved in DME was principally employed as the electrolyte for testing, while 1 m KPF_6_ dissolved in EC/PC and 1 m KFSI dissolved in DME were also used for comparison. In terms of SIBs, an electrolyte composed of 1 m NaPF_6_ dissolved in DME was employed. Notably, the integrated Cu_2_Se electrode used to assemble SIBs was first fetched out from the PIB, and then washed by DME solvent to remove the residual KPF_6_ salt. Galvanostatic tests were carried out on a Land‐2100 automatic battery tester. CV and EIS were performed on a VSP multichannel potentiostatic‐galvanostatic system (Biologic, France).

### Computational Details

The solvation structures of K^+^−solvent−anion complexes, KPF_6_ or KFSI in DME or EC/PC electrolyte, were simulated through the molecular dynamics (MD) simulations. All simulations were performed with GROMACS package.^[^ ^48^, ^49^, ^50^ ^]^ The empirical force field was mainly obtained from AMBER force field,^[^ ^51^, ^52^, ^53^ ^]^ and these simulations were performed at constant temperature and volume with V‐rescale coupling.^[^ ^54^ ^]^ The Lorentz–Berthelot rules were used to obtain the inter‐molecular Lennard–Jones parameters and the cut‐off for the Vdw interaction was set at 10 Å. The box size was set as 15 × 15 × 15 nm^3^. After 10 ns NVT simulation at 300 K, they were assembled as a stable solvation complex. In the simulations, the volume and target temperature (including 250, 300, and 500 K) in each MD simulation were set. The initial pressures were vary from 1 to 2 atm. Consequently, the amount of added electrolytes can be calculated according to the initial pressure of the system. In each simulation, the system was first equilibrated at 500 K for 5 ns, and then gradually annealed to the target temperature in 4 ns, followed by another 5 ns equilibration at the target temperature and 1 atm. Another 15 ns NVT simulation using the Nose–Hoover thermostat was performed for each system, in which the coordinates for all the atoms were accumulated every 10 ns for analysis.^[^ ^55^ ^]^ To fully equilibrate the system, the NPT simulations were performed at 300 K and 1 atm for 2 ns, and the system was further quenched from 1000 to 300 K in NVT ensemble for 10 ns. The above process has been done for three times. Then, the system was equilibrated in NPT ensemble at 300 K and 1 atom for 2 ns, and the last 1 ns trajectory was used to calculate the averaged volume for fully comparison.

The structural optimization of the most probable distribution of K^+^−solvent−anion complex was performed using the PBE0 level of DFT with the Def2TZVP basis set.^[^ ^56^, ^57^, ^58^ ^]^ Additionally, Se_13_ cluster was extracted from the trigonal selenium structure as the most stable unit (compared to a dozen other cases) to evaluate the interaction between the precursory selenium electrode and the electrolyte.^[^ ^59^ ^]^ And Grimme's DFT‐D2 dispersion correction method was employed to describe the vdW interactions.^[^ ^60^, ^61^ ^]^ The HOMO, the lowest unoccupied molecular orbitals, and the electrostatic potentials of the optimized geometries were plotted using the Gaussview 6.0 tool.^[^ ^62^ ^]^

## Conflict of Interest

The authors declare no conflict of interest.

## Supporting information

Supporting Information

## Data Availability

Research data are not shared.

## References

[1] N. Yabuuchi , K. Kubota , M. Dahbi , S. Komaba , Chem. Rev. 2014, 114, 11636. 25390643 10.1021/cr500192f

[2] J.‐Y. Hwang , S.‐T. Myung , Y.‐K. Sun , Adv. Funct. Mater. 2018, 28, 1802938.

[3] Y. Jiang , Y. Wang , J. Ni , L. Li , InfoMat. 2021, 3, 339.

[4] Y. Sun , S. Guo , H. Zhou , Adv. Energy Mater. 2019, 9, 1800212.

[5] H. Kim , J. C. Kim , M. Bianchini , D.‐H. Seo , J. Rodriguez‐Garcia , G. Ceder , Adv. Energy Mater. 2018, 8, 1702384.

[6] Y. Xia , W. Jin , Y. Qi , H. Li , Z. Jian , W. Chen , Chin. Chem. Lett. 2021, 32, 2433.

[7] Z. Hu , Q. Liu , S.‐L. Chou , S.‐X. Dou , Adv. Mater. 2017, 29, 1700606. 10.1002/adma.201700606 28643429

[8] W. Wang , B. Jiang , C. Qian , F. Lv , J. Feng , J. Zhou , K. Wang , C. Yang , Y. Yang , S. Guo , Adv. Mater. 2018, 30, 1801812. 10.1002/adma.201801812 29894007

[9] L. Yang , W. Hong , Y. Tian , G. Zou , H. Hou , W. Sun , X. Ji , Chem. Eng. J. 2020, 385, 123838.

[10] Y. Liu , Q. Feng , W. Liu , Q. Li , Y. Wang , B. Liu , L. Zheng , W. Wang , L. Huang , L. Chen , X. Xiong , Y. Lei , Nano Energy 2021, 81, 105641.

[11] J. Sun , Z. Du , Y. Liu , W. Ai , K. Wang , T. Wang , H. Du , L. Liu , W. Huang , Adv. Mater. 2021, 33, 2003845. 10.1002/adma.202003845 33491836

[12] J. Zhou , Y. Liu , S. Zhang , T. Zhou , Z. Guo , InfoMat. 2020, 2, 437.

[13] G. Fang , Q. Wang , J. Zhou , Y. Lei , Z. Chen , Z. Wang , A. Pan , S. Liang , ACS Nano 2019, 13, 5635. 31022345 10.1021/acsnano.9b00816

[14] X. Zhou , P. Gao , S. Sun , D. Bao , Y. Wang , X. Li , T. Wu , Y. Chen , P. Yang , Chem. Mater. 2015, 27, 6730.

[15] C. Wang , Q. Hu , Y. Wei , D. Fang , W. Xu , Z. Luo , Ionics 2017, 23, 3571.

[16] H. Wang , X. Lan , D. Jiang , Y. Zhang , H. Zhong , Z. Zhang , Y. Jiang , J. Power Sources 2015, 283, 187.

[17] N. Shi , Y. Chu , B. Xi , M. Huang , W. Chen , B. Duan , C. Zhang , J. Feng , S. Xiong , Adv. Energy Mater. 2020, 10, 2002298.

[18] K. Zhang , M. Park , L. Zhou , G.‐H. Lee , W. Li , Y.‐M. Kang , J. Chen , Adv. Funct. Mater. 2016, 26, 6728.

[19] K. Zhang , Z. Hu , X. Liu , Z. Tao , J. Chen , Adv. Mater. 2015, 27, 3305. 25899537 10.1002/adma.201500196

[20] H. Fan , H. Yu , X. Wu , Y. Zhang , Z. Luo , H. Wang , Y. Guo , S. Madhavi , Q. Yan , ACS Appl. Mater. Interfaces 2016, 8, 25261. 27559752 10.1021/acsami.6b07300

[21] G. D. Park , Y. C. Kang , Chem. ‐ Eur. J. 2016, 22, 4140.26864320

[22] S.‐K. Park , J.‐S. Park , Y. C. Kang , J. Mater. Chem. A 2018, 6, 1028.

[23] Z. Zhang , X. Shi , X. Yang , Y. Fu , K. Zhang , Y. Lai , J. Li , ACS Appl. Mater. Interfaces 2016, 8, 13849. 27218287 10.1021/acsami.5b12148

[24] A. Wang , W. Hong , L. Li , R. Guo , Y. Xiang , Y. Ye , G. Zou , H. Hou , X. Ji , Energy Storage Mater. 2022, 44, 145.

[25] H. Tian , H. Tian , S. Wang , S. Chen , F. Zhang , L. Song , H. Liu , J. Liu , G. Wang , Nat. Commun. 2020, 11, 5025. 33024100 10.1038/s41467-020-18820-y PMC7538427

[26] S. Kim , M. Cho , Y. Lee , Adv. Energy Mater. 2020, 10, 1903477.

[27] Y. Huang , Z. Wang , M. Guan , F. Wu , R. Chen , Adv. Mater. 2020, 32, 2003534. 10.1002/adma.202003534 32844532

[28] X. Gu , T. Tang , X. Liu , Y. Hou , J. Mater. Chem. A 2019, 7, 11566.

[29] H. Wang , Y. Jiang , A. Manthiram , Adv. Energy Mater. 2018, 8, 1701953.

[30] X. Yang , H. Wang , D. Y. W. Yu , A. L. Rogach , Adv. Funct. Mater. 2018, 28, 1706609.

[31] B. Kalimuthu , K. Nallathamby , ACS Appl. Mater. Interfaces 2017, 9, 26756. 28718630 10.1021/acsami.7b05103

[32] J. Ding , H. Zhou , H. Zhang , T. Stephenson , Z. Li , D. Karpuzov , D. Mitlin , Energy Environ. Sci. 2017, 10, 153.

[33] J. Zhang , Z. Li , X. W. Lou , Angew. Chem., Int. Ed. 2017, 56, 14107. 10.1002/anie.201708105 28914479

[34] P. Ge , S. Li , L. Xu , K. Zou , X. Gao , X. Cao , G. Zou , H. Hou , X. Ji , Adv. Energy Mater. 2019, 9, 1803035.

[35] A. Du , Y. Zhao , Z. Zhang , S. Dong , Z. Cui , K. Tang , C. Lu , P. Han , X. Zhou , G. Cui , Energy Storage Mater. 2020, 26, 23.

[36] Q. Liu , W. Deng , Y. Pan , C.‐F. Sun , Chem. Sci. 2020, 11, 6045. 34094097 10.1039/d0sc01474e PMC8159323

[37] Y. Liu , Z. Tai , Q. Zhang , H. Wang , W. K. Pang , H. K. Liu , K. Konstantinov , Z. Guo , Nano Energy 2017, 35, 36.

[38] Y. Cui , A. Abouimrane , J. Lu , T. Bolin , Y. Ren , W. Weng , C. Sun , V. A. Maroni , S. M. Heald , K. Amine , J. Am. Chem. Soc. 2013, 135, 8047. 23631402 10.1021/ja402597g

[39] J. Ming , Z. Cao , Q. Li , W. Wahyudi , W. Wang , L. Cavallo , K.‐J. Park , Y.‐K. Sun , H. N. Alshareef , ACS Energy Lett. 2019, 4, 1584.

[40] J. Zhang , Z. Cao , L. Zhou , G.‐T. Park , L. Cavallo , L. Wang , H. N. Alshareef , Y.‐K. Sun , J. Ming , ACS Energy Lett. 2020, 5, 3124.

[41] J. Zhang , Z. Cao , L. Zhou , G. Liu , G.‐T. Park , L. Cavallo , L. Wang , H. N. Alshareef , Y.‐K. Sun , J. Ming , ACS Energy Lett. 2020, 5, 2651.

[42] K. Kubota , M. Dahbi , T. Hosaka , S. Kumakura , S. Komaba , Chem. Rec. 2018, 459. 29442429 10.1002/tcr.201700057

[43] J. Wang , B. Wang , X. Liu , J. Bai , H. Wang , G. Wang , Chem. Eng. J. 2020, 382, 123050.

[44] N. Hussain , S. Zeng , Z. Feng , Y. Zhai , C. Wang , M. Zhao , Y. Qian , L. Xu , Nano Res. 2021, 14, 3552.

[45] J. Zhao , X. Yang , S. Li , N. Chen , C. Wang , Y. Zeng , F. Du , CCS Chem. 2020, 2, 2498.

[46] Y. Li , X. Sun , Z. Cheng , X. Xu , J. Pan , X. Yang , F. Tian , Y. Li , J. Yang , Y. Qian , Energy Storage Mater. 2019, 22, 275.

[47] H. Peng , J. Ren , Y. Wang , Y. Xiong , Q. Wang , Q. Li , X. Zhao , L. Zhan , L. Zheng , Y. Tang , Y. Lei , Nano Energy 2021, 88, 106307.

[48] H. Bekker , H. J. C. Berendsen , E. J. Dijkstra , S. Achterop , R. Vondrumen , D. Vanderspoel , A. Sijbers , H. Keegstra , B. Reitsma , M. K. R. Renardus , in Physics Computing '92, (Eds: R. A. DeGroot , J. Nadrchal ), World Scientific, Singapore 1993, pp. 252–256.

[49] D. Van Der Spoel , E. Lindahl , B. Hess , G. Groenhof , A. E. Mark , H. J. C. Berendsen , J. Comput. Chem. 2005, 26, 1701. 16211538 10.1002/jcc.20291

[50] B. Hess , C. Kutzner , D. van der Spoel , E. Lindahl , J. Chem. Theory Comput. 2008, 4, 435. 26620784 10.1021/ct700301q

[51] D. Bassolino‐Klimas , H. E. Alper , T. R. Stouch , J. Am. Chem. Soc. 1995, 117, 4118.

[52] J. Ming , Z. Cao , Y. Wu , W. Wahyudi , W. Wang , X. Guo , L. Cavallo , J.‐Y. Hwang , A. Shamim , L.‐J. Li , Y.‐K. Sun , H. N. Alshareef , ACS Energy Lett. 2019, 4, 2613.

[53] J. Wang , R. M. Wolf , J. W. Caldwell , P. A. Kollman , D. A. Case , J. Comput. Chem. 2004, 25, 1157. 15116359 10.1002/jcc.20035

[54] G. Bussi , D. Donadio , M. Parrinello , J. Chem. Phys. 2007, 126, 014101. 17212484 10.1063/1.2408420

[55] S. Melchionna , G. Ciccotti , B. L. Holian , Mol. Phys. 1993, 78, 533.

[56] C. Adamo , V. Barone , J. Chem. Phys. 1999, 110, 6158.

[57] J. P. Perdew , K. Burke , M. Ernzerhof , Phys. Rev. Lett. 1996, 77, 3865. 10062328 10.1103/PhysRevLett.77.3865

[58] F. Weigend , R. Ahlrichs , Phys. Chem. Chem. Phys. 2005, 7, 3297. 16240044 10.1039/b508541a

[59] Y. Wang , Z. Zhu , Z. Sun , Q. Hu , J. Li , J. Jiang , X. Huang , Chem. Eur. J. 2020, 26, 1624. 31971636 10.1002/chem.201904256

[60] S. Grimme , J. Comput. Chem. 2006, 27, 1787. 16955487 10.1002/jcc.20495

[61] S. Grimme , J. Antony , S. Ehrlich , H. Krieg , J. Chem. Phys. 2010, 132, 154104. 20423165 10.1063/1.3382344

[62] R. Dennington , T. A. Keith , J. M. Millam , SemichemInc. Shawnee Mission KS 2016.

