# Supplementary material for: In Situ Fabrication of Cuprous Selenide Electrode via Selenization of Copper Current Collector for High‐Efficiency Potassium‐Ion and Sodium‐Ion Storage
Source: Adv Sci (Weinh). 2021 Dec 23;9(5):2104630. doi: 10.1002/advs.202104630 (PMC8844570; doi:10.1002/advs.202104630)

ADVANCED SCIENCE
Open Access

Supporting Information

for *Adv. Sci.*, DOI: 10.1002/advs.202104630

In-situ Fabrication of Cuprous Selenide Electrode via Selenization of Copper Current Collector for High-Efficiency Potassium-Ion and Sodium-Ion Storage

Xi Chen, Malin Li, Shi-Ping Wang, Chunzhong Wang, Zexiang Shen, Fu-Quan Bai*, and Fei Du**

Supporting Information

In-situ Fabrication of Cuprous Selenide Electrode via Selenization of Copper Current Collector for High-Efficiency Potassium-Ion and Sodium-Ion Storage

Xi Chen, Malin Li, Shi-Ping Wang, Chunzhong Wang, Zexiang Shen, Fu-Quan Bai*, and Fei Du**

X. Chen, Prof. C. Wang, Prof. F. Du
Key Laboratory of Physics and Technology for Advanced Batteries (Ministry of Education), State Key Laboratory of Superhard Materials, College of Physics, Jilin University, Changchun, 130012, People's Republic of China
E-mail: dufei@jlu.edu.cn

Dr. M. Li
State Key Laboratory of Inorganic Synthesis and Preparative Chemistry, College of Chemistry, Jilin University, Changchun, 130012, People's Republic of China
E-mail: malinl@jlu.edu.cn

S.-P. Wang, Prof. F.-Q. Bai
Laboratory of Theoretical and Computational Chemistry, Institute of Theoretical Chemistry and College of Chemistry, Jilin University, Changchun, 130012, People's Republic of China
E-mail: baifq@jlu.edu.cn

Prof. Z. Shen
Division of Physics and Applied Physics, School of Physical and Mathematical Sciences, Nanyang Technological University, Singapore, 637616, Singapore

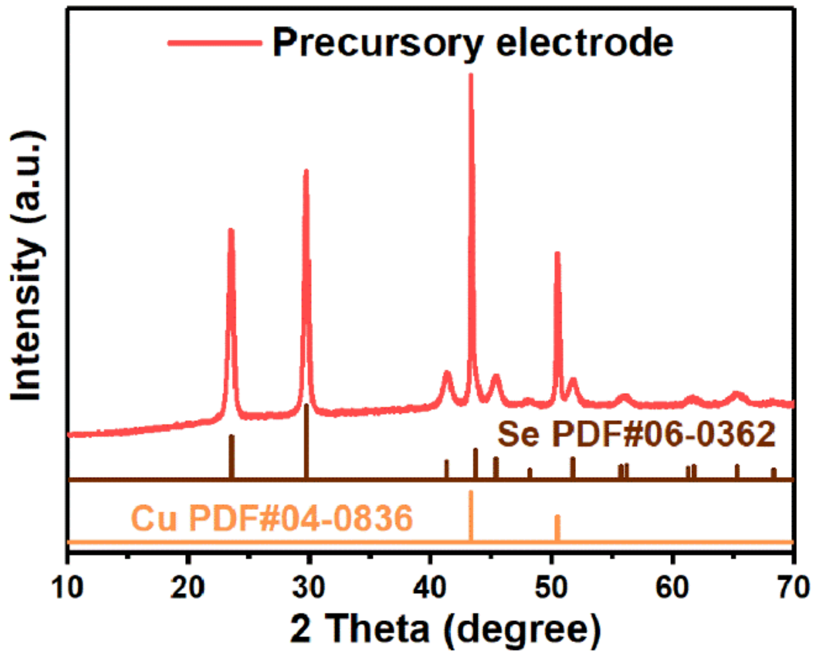

Figure S1. XRD pattern of the precursory electrode.

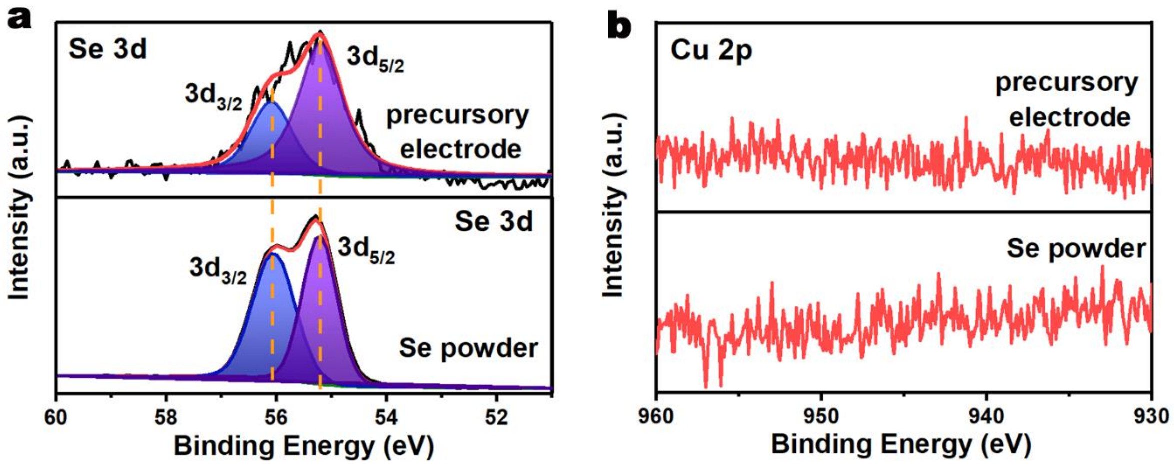

Figure S2. (a) Se 3d and (b) Cu 2p spectra of the precursory electrode and Se powder.

As shown in the high-resolution Se 3d spectrum of the precursory electrode (Figure S2a), the two peaks centered at 55.2 and 56.0 eV correspond to the $3d_{5/2}$ and $3d_{3/2}$ states of elemental Se, which are in accordance with those of the Se powder. In addition, no signal of Cu could be observed in Cu 2p spectra of both the Se powder and the precursory electrode, in which the Cu foil was total covered by commercial Se, further demonstrating the stability of Se against the Cu foil in the as-prepared precursory electrode.

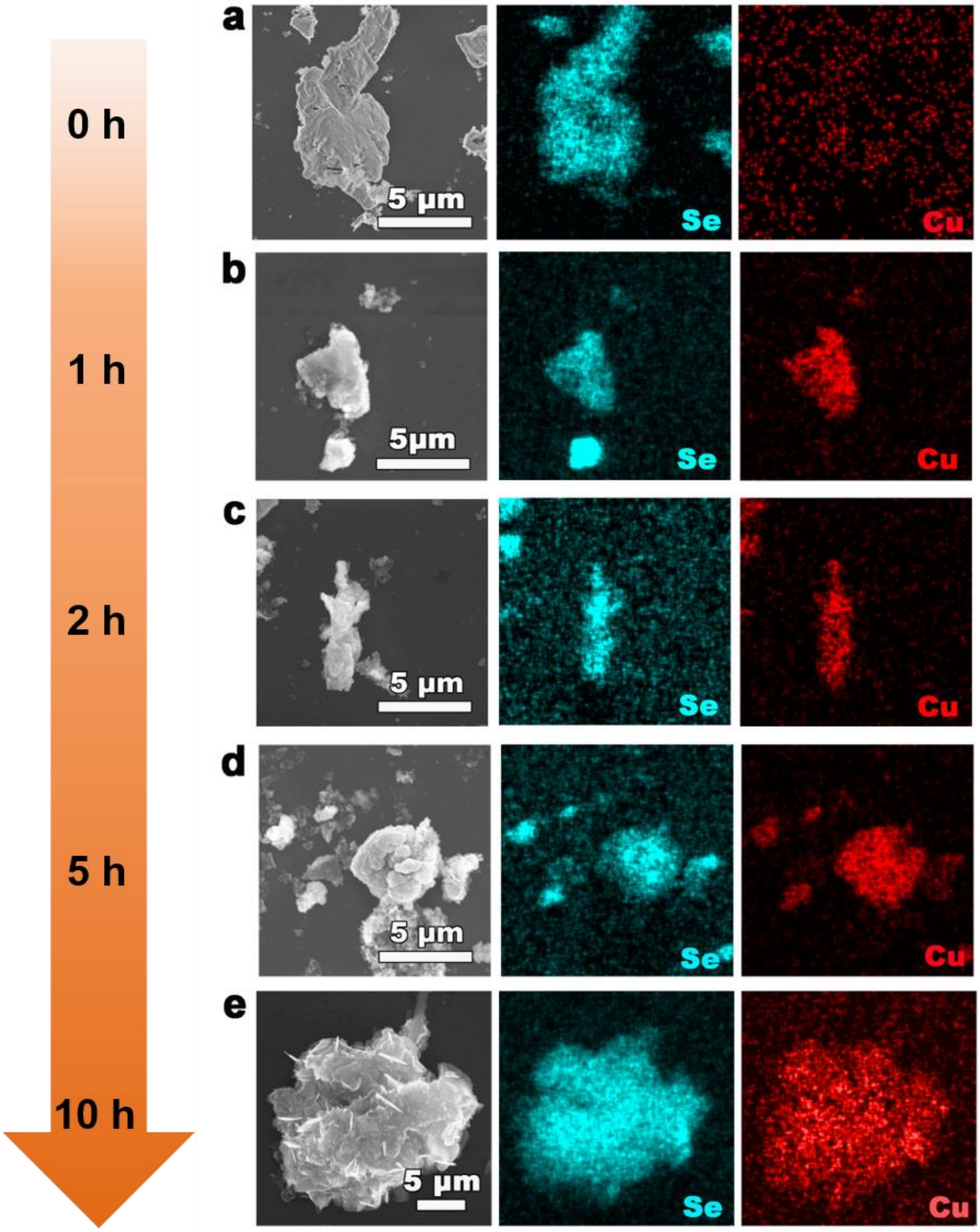

Figure S3. Element mapping of the electrodes relaxed for 0, 1, 2, 5, and 10 h.

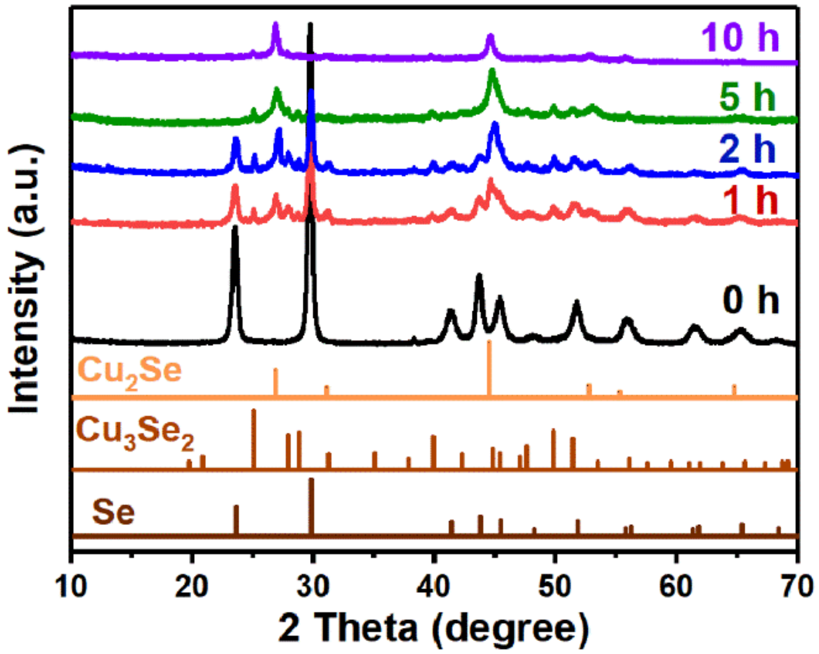

Figure S4. XRD pattern of the electrodes relaxed for 0, 1, 2, 5, and 10 h**.**

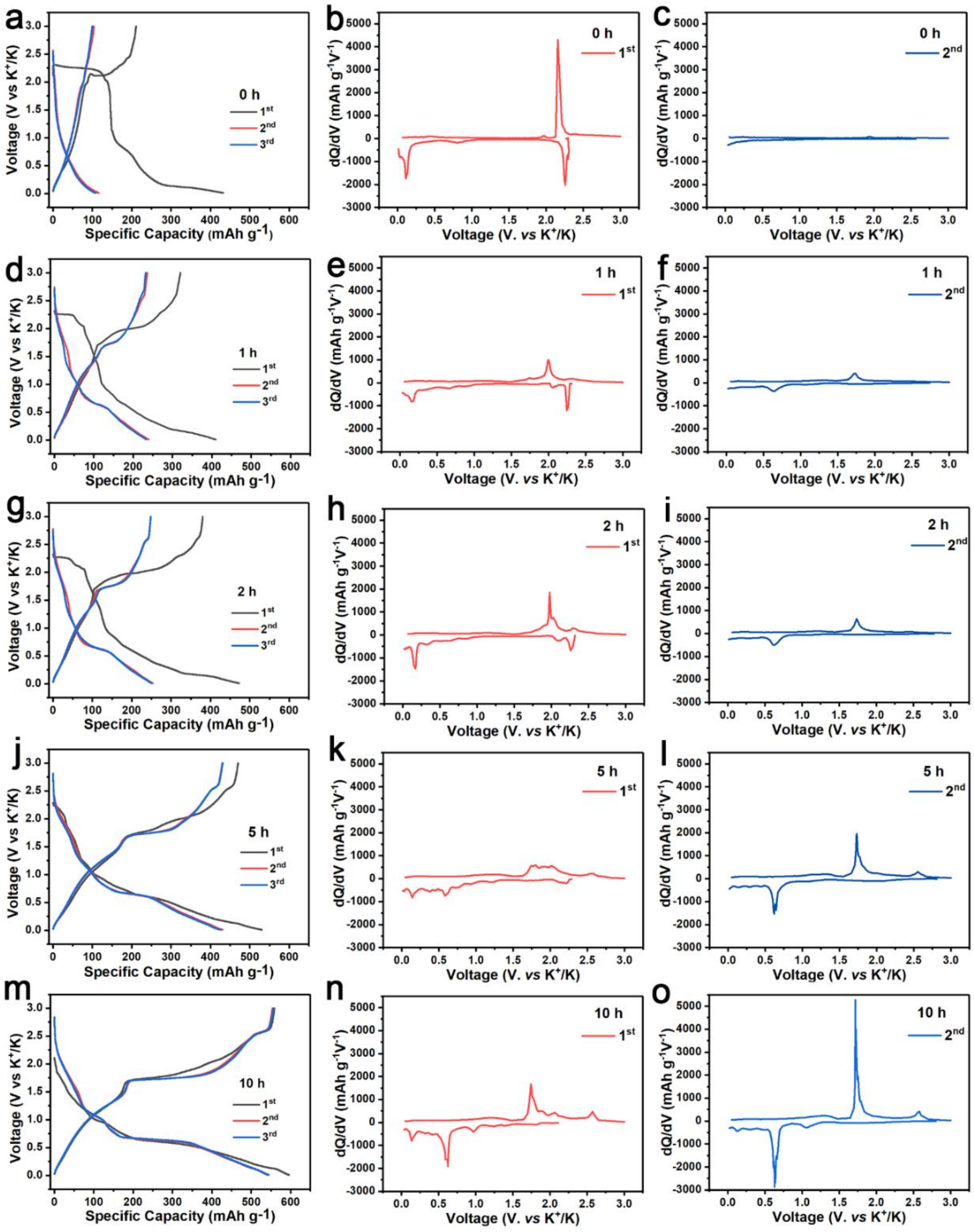

Figure S5. The galvanostatic charge and discharge profiles and the corresponding dQ/dV plots of the batteries with the relaxation period of 0, 1, 2, 5, and 10 h.

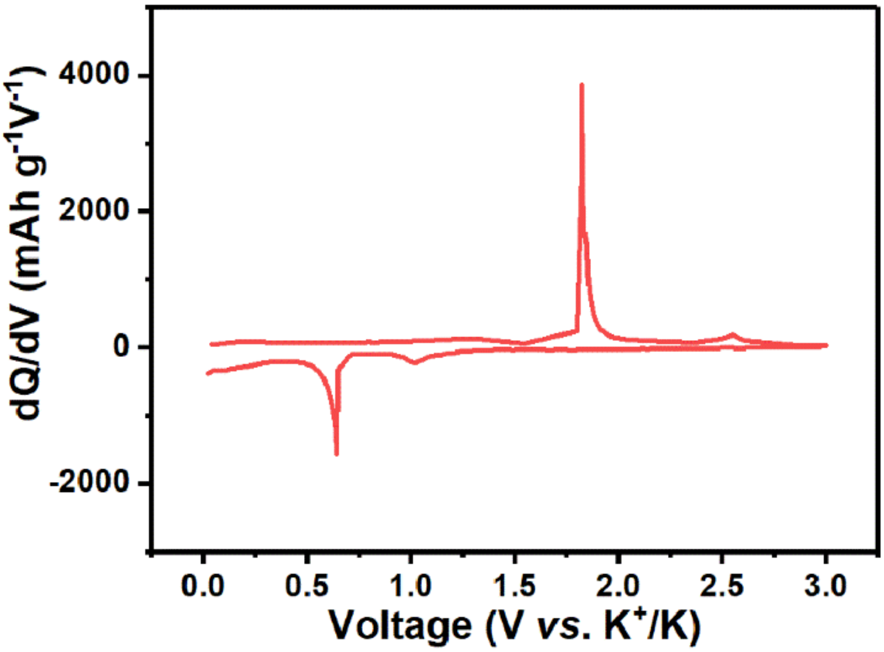

Figure S6. The dQ/dV plots of the battery with commercial cuprous selenide as the electrode.

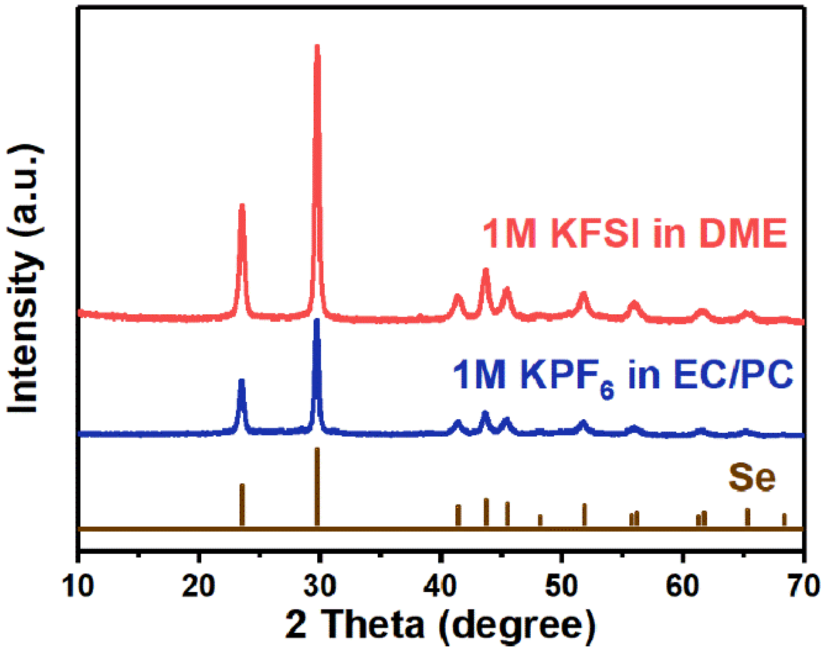

Figure S7. XRD patterns of the electrodes relaxed for 10 h in the batteries with the electrolytes of 1 M KFSI in DME and 1 M KPF_6 in EC/PC.

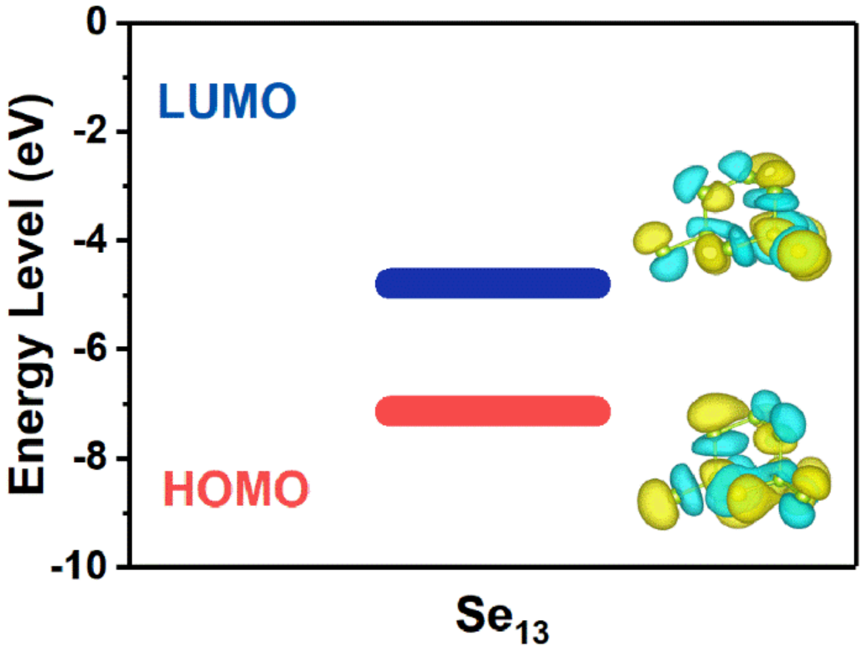

Figure S8. The HOMO and LUMO energy levels of Se_{13} cluster (inset: the corresponding molecular orbital profiles).

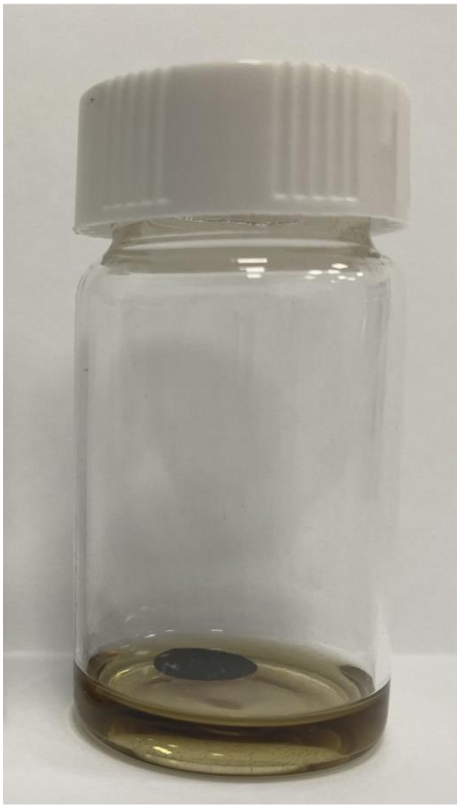

Figure S9. The digital picture of the solution used to immerse the electrode relaxed for 10 h in KFSI/DME electrolyte.

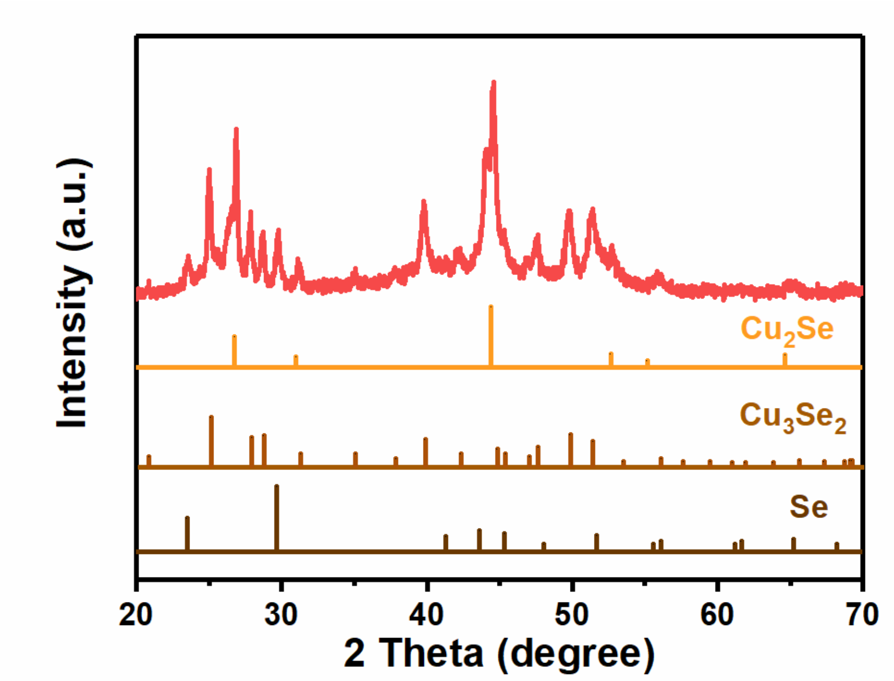

Figure S10. XRD pattern of the electrode relaxed for 10 h in assembled SIBs with $NaPF_6$/DME electrolyte.

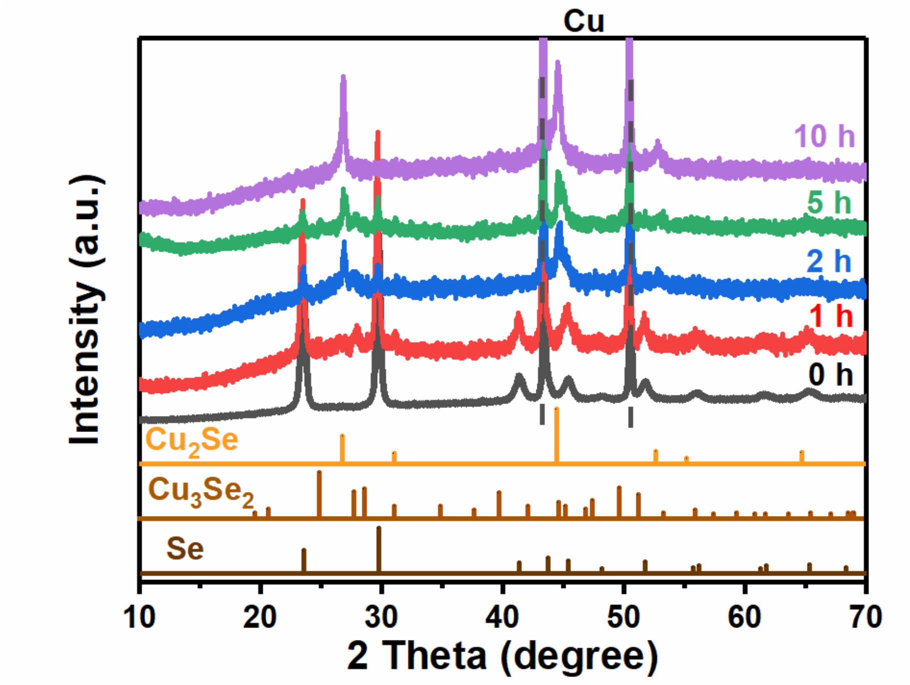

Figure S11. XRD patterns of the precursory electrodes with lower mass loading after being relaxed for 0, 1, 2, 5, and 10 h.

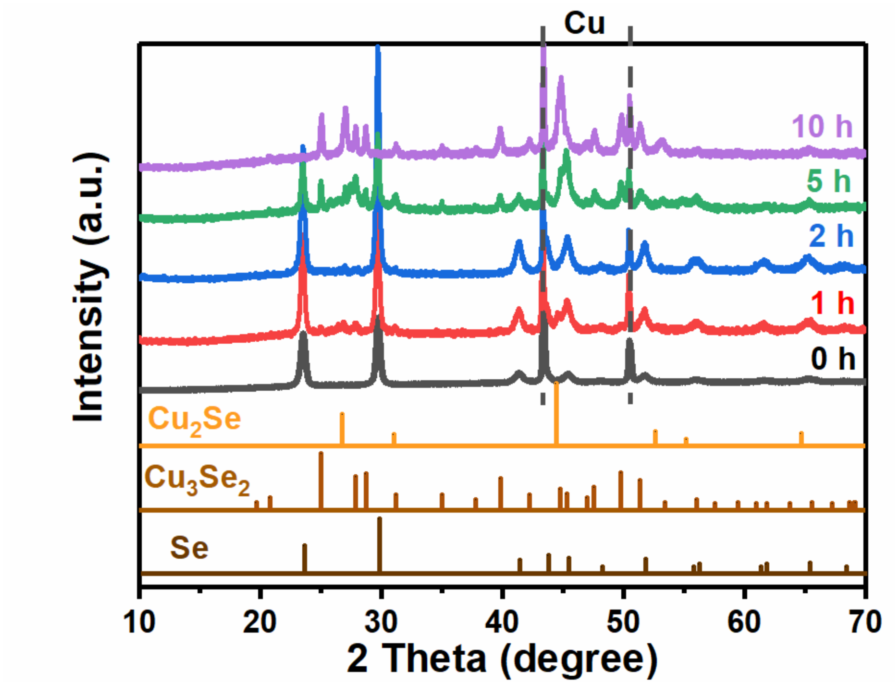

Figure S12. XRD patterns of the precursory electrodes with higher mass loading after being relaxed for 0, 1, 2, 5, and 10 h.

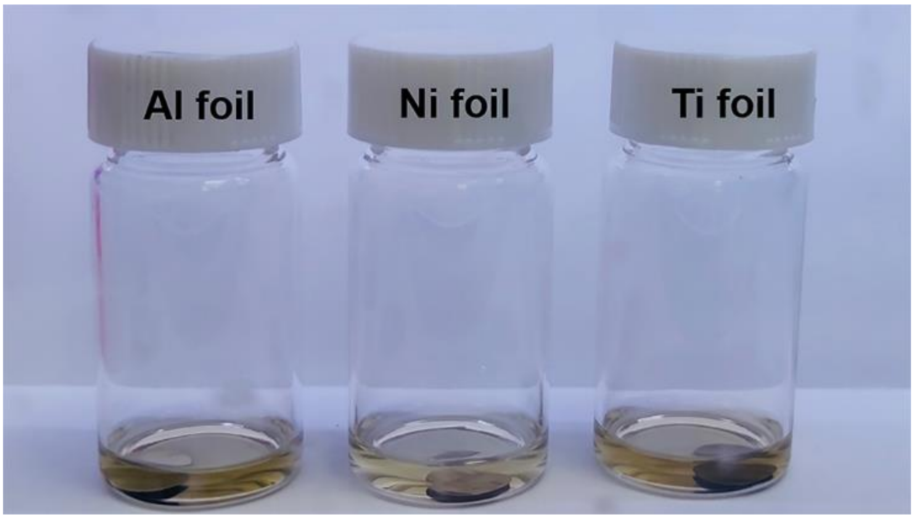

Figure S13. The digital pictures of the solution used to immerse the electrodes with Al, Ni, and Ti current collectors.

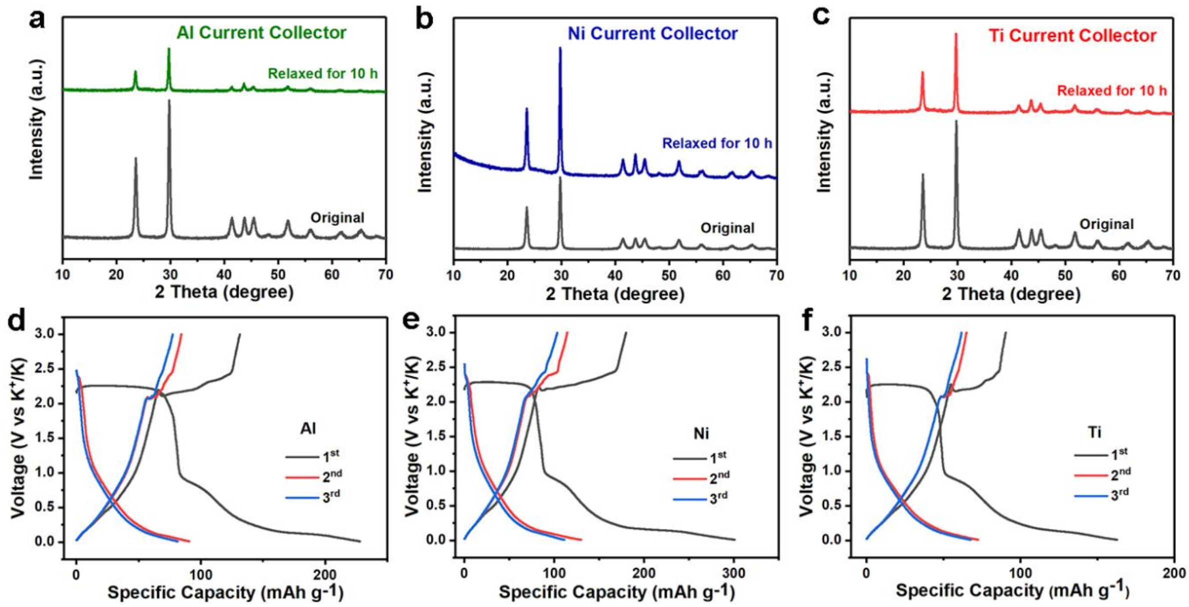

Figure S14. (a–c) XRD patterns of the electrodes using Al, Ni, and Ti as the current collector before and after being relaxed for 10 h. (d–f) Galvanostatic charge and discharge profiles of the batteries using Al, Ni, and Ti as the current collector at 0.2 A g^{-1}.

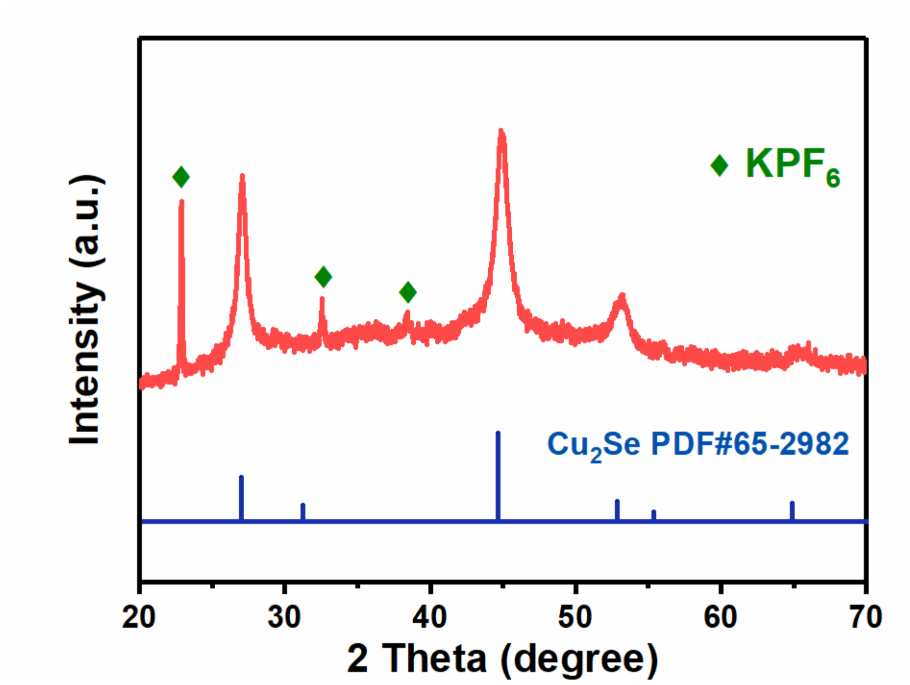

Figure S15. XRD pattern of the integrated Cu_2Se electrode after cycling in PIBs.

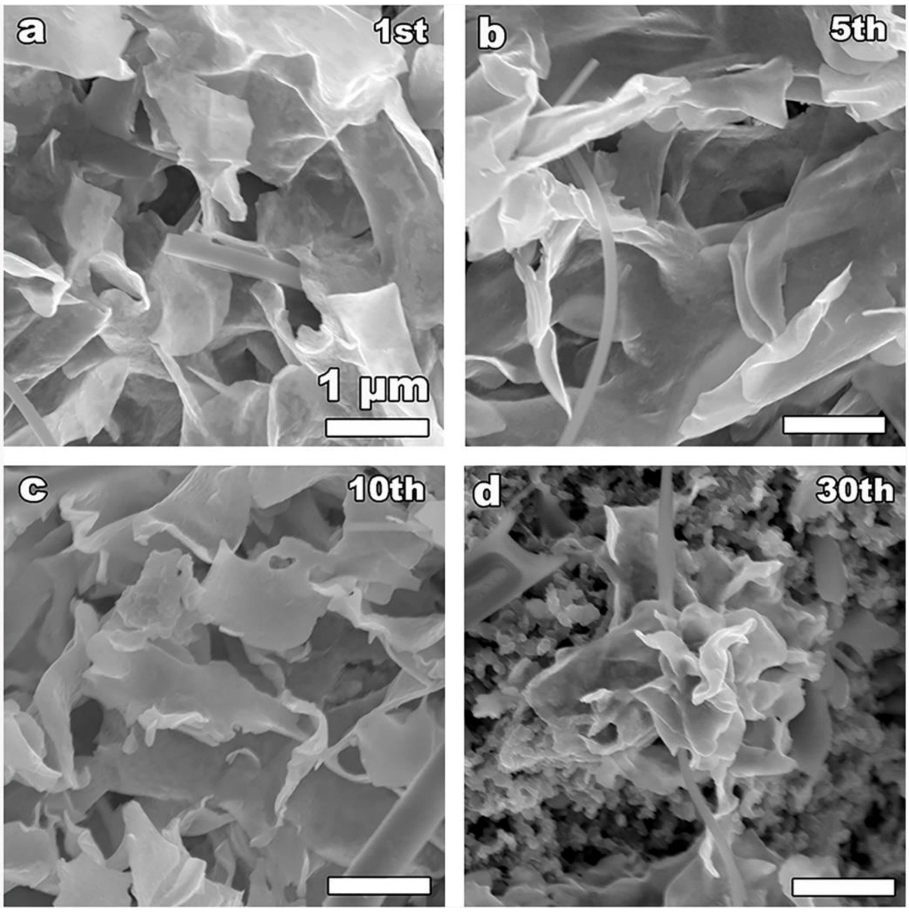

Figure S16. Ex-situ SEM images of the integrated Cu_2Se electrode after the 1st, 5th, 10th, and 30th cycles in PIBs.

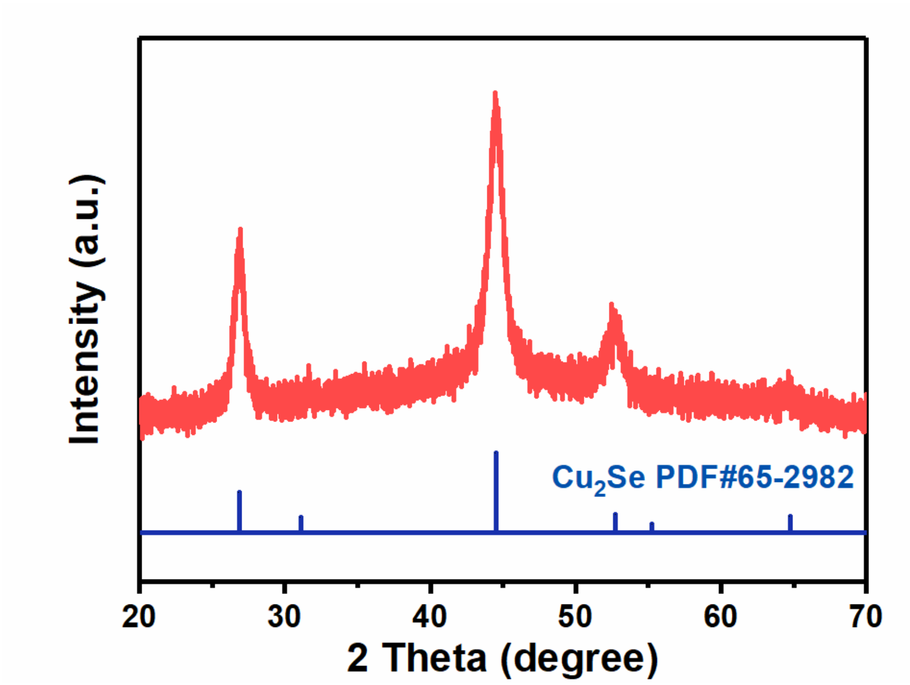

Figure S17. XRD pattern of the integrated Cu_2Se electrode after cycling in SIBs.

Table S1. The fitted resistances in equivalent circuits for the integrated Cu_2S electrode in PIBs. The equivalent circuits is shown below the Table, where R_s is the series resistance of the electrodes, electrolyte and the current collectors, R_{ct} is the charge-transfer resistance, CPE is the constant phase element, and W is the Warburg impedance.

	**R_s (Ω)**	**R_{ct} (Ω)**
**OCV**	4.6	2.2
**1st**	5.1	5.4
**10th**	6.7	17.4
**50th**	8.3	18.7

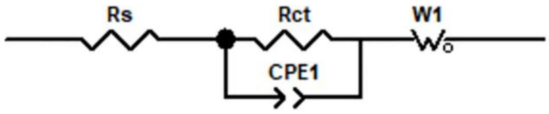

Table S2. The fitted resistances in equivalent circuits for the integrated Cu_2S electrode in SIBs. The equivalent circuits are shown below the Table, where R_s is the series resistance of the electrodes, electrolyte and the current collectors, R_{ct} is the charge-transfer resistance, CPE is the constant phase element, and W is the Warburg impedance.

	**R_s (Ω)**	**R_{ct} (Ω)**
**OCV**	1.9	3.3
**1st**	2.0	1.5
**10th**	3.4	1.5
**50th**	2.7	3.3

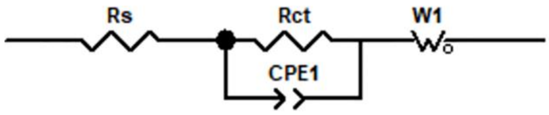

Supplement: Supplementary file 1 — Supporting Information [file ADVS-9-2104630-s001.pdf]
